# Influence of nanofibers on growth and gene expression of human tendon derived fibroblast

**DOI:** 10.1186/1475-925X-9-9

**Published:** 2010-02-17

**Authors:** Christina Theisen, Susanne Fuchs-Winkelmann, Karola Knappstein, Turgay Efe, Jan Schmitt, Juergen RJ Paletta, Markus D Schofer

**Affiliations:** 1 Department of Orthopaedics and Rheumatology, University of Marburg, Baldingerstrasse, 35043 Marburg, Germany

## Abstract

**Background:**

Rotator cuff tears are a common and frequent lesion especially in older patients. The mechanisms of tendon repair are not fully understood. Common therapy options for tendon repair include mini-open or arthroscopic surgery. The use of growth factors in experimental studies is mentioned in the literature. Nanofiber scaffolds, which provide several criteria for the healing process, might be a suitable therapy option for operative treatment. The aim of this study was to explore the effects of nanofiber scaffolds on human tendon derived fibroblasts (TDF's), as well as the gene expression and matrix deposition of these fibroblasts.

**Methods:**

Nanofibers composed of PLLA and PLLA/Col-I were seeded with human tendon derived fibroblasts and cultivated over a period of 22 days under growth-inductive conditions, and analyzed during the course of culture, with respect to gene expression of different extra cellular matrix components such as collagens, bigylcan and decorin. Furthermore, we measured cell densities and proliferation by using fluorescene microscopy.

**Results:**

PLLA nanofibers possessed a growth inhibitory effect on TDF's. Furthermore, no meaningful influence on the gene expression of collagen I, collagen III and decorin could be observed, while the expression of collagen X increased during the course of cultivation. On the other hand, PLLA/Col-I blend nanofibers had no negative influence on the growth of TDF's. Furthermore, blending PLLA nanofibers with collagen had a positive effect on the gene expression of collagen I, III, X and decorin. Here, gene expression indicated that focal adherence kinases might be involved.

**Conclusion:**

This study indicates that the use of nanofibers influence expression of genes associated with the extra cellular matrix formation. The composition of the nanofibers plays a critical role. While PLLA/Col-I blend nanofibers enhance the collagen I and III formation, their expression on PLLA nanofibers was more comparable to controls. However, irrespective of the chemical composition of the fibres, the collagen deposition was altered, an effect which might be associated with a decreased expression of biglycanes.

## Background

The rotator cuff is a muscle coat that encloses the shoulder and with its four parts, is responsible for the movement and integrity of the glenohumeral joint. Tears mainly occur in the supraspinatus tendon [1,2]. In common literature, the frequency of rotator cuff tears in anatomical studies varies between 17% and 72% [3,4]. The appearance of a rotator cuff lesion is related to and increases with the patient' age [5]. In 1911, E. A. Codman published the first successful rotator cuff refixation after open repair of a supraspinatus tendon using silk sutures [6]. Compared to other injuries, rotator cuff tears show no tendency towards healing, so that operative surgery is necessary in most cases. Rerupture due to, osteoporoses, poor vascularization, degenerative changings such as atrophy and fatty degeneration of the muscle or the size of the original tear contribute to the high failure rate [7-9]. Because of this, there is strong clinical relevance towards methods which improve rotator cuff tendon healing. It was also shown that rotator cuff healing occurs by reactive scar formation rather than regeneration of a histologically normal insertion zone [10].

Tendons and ligaments are very similar connective tissue. They are an essential component of the musculoskeletal system. They provide stability and movement of joints. The strength of tendons and ligaments varies depending on anatomic request and condition and they are able to adapt to various conditions [11]. There are few specific biochemical markers known for tendons and ligaments [12,13].

The current literature provides promising results with alternative methods for tendon repair using allogeneic or xenogeneic grafts such as collagen-rich dermis and small intestinal submucosa [14-17]. Besides tissue scaffolds the local application of growth factors such as fibroblast growth factor-2 improves the rotator cuff repair and accelerates the initial tendon-to-bone healing [18,19]. Seeherman et al. showed that delivery of recombinant human bone morphogenetic protein-12 (rhBMP-12) in a collagen or hyaluronan sponge resulted in accelerated healing of acute full-thickness rotator cuff repairs in a sheep model [20].

In further studies, we showed that poly(l-lactic acid) (PLLA) and collagen-I (Col-I) electrospun nanofibers are applicable grafts for the reconstruction of large bony defects by promoting growth and osteogenetic differentiation of stem cells [21,22]. In this study we focused on the effects of nanofiber scaffolds on human tendon derived fibroblasts (TDF's), gene expression and matrix deposition of these fibroblasts.

Therefore, an ideal scaffold for tendon repair should match several criteria. It has to be tolerated by the tenocytes, it must facilitate the colonialisation (promoting either migration or proliferation of the cells) and furthermore, it must enhance the formation of the extra cellular matrix (ECM) during the healing process. Here, scaffolds based on electrospun nanofibers, offer great advantages. Such matrices show morphological similarities to the natural ECM, characterized by ultrafine continuous fibers, high surface-to-volume ratio, high porosity and variable pore-size distribution [23]. These nanofibers can be produced by a broad spectrum of polymers including biocompatible as well as biodegradable polymers, such as poly(glycolic acid) (PGA), PLLA, poly-ε caprolactone (PCL), polyurethanes, polyphosphazenes, collagen, gelatin, and chitosan as well as copolymers from the corresponding monomers in various compositions [24,25]. This allows the production of a broad spectrum of nanofiber based scaffolds with different mechanical and biophysical properties. Depending on the polymer the nanofibers were tolerated by a variety of cell types including human mesencymal stem cells (hMSC) and TDF's.

## Methods

### Construction of nanofibers and characterization

A 4.5% (w/v) PLLA (Resomer L210, Boehringer Ingelheim Germany) solution in hexafluoroisopropanol (HFIP) was prepared at room temperature by stirring overnight until a homogenous solution was obtained. Spinning process was performed at a flow rate of 14 μl/min with an applied voltage of 10 - 18 kV and a distance of 15 cm.

The PLLA collagen-I (PLLA/Col-I) blend nanofibers with a polymer ratio of 4:1 were fabricated as reported earlier [22].

For cell culture experiments, all samples of nonwoven nanofibers were prepared under aseptic conditions and collected on 19 mm cover slips.

Static contact angles of water were measured using the sessile drop method with a G10 Drop Shape Analysis System (Krüss, Hamburg, Germany) and calculated using Data Physics SCA20 Contact Angle Analyzer Software. For scanning electron microscopy (SEM), samples were splutter-coated with gold in an AUTO-306 (BOC Edwards, Crawley, Sussex, U.K.) high-vacuum coating system and examined in a SEM (S-4100, Hitachi Ltd., Tokyo, Japan) at an accelerating voltage of 5 kV in the SE mode.

### Human tendon derived fibroblasts: cell isolation and culture

TDF's were obtained from consenting patients with the approval of the institutional review board. The indication for surgery with tenotomy of the long biceps tendon was instability of the tendon, tendonitis or incomplete rupture of the long biceps tendon. The routinely removed tendon was cut into pieces of approximately 5 mm and subjected to collagenase digestion for a period of 30 min at 37°C. After removal of the collagenase, pieces were rinsed with phosphate puffered saline (PBS) and explanted to culture flasks in Dulbecco' modified eagles medium (DMEM) containing 10% fetal calf serum (FCS) and 1% penicillin/streptomycin. Within 1 week - when cells migrated from the tendon and became attached to the culture flask - tendon pieces were removed and the cells were further cultured to confluence.

For experiments, TDF's (passage 2) were seeded at a density of 3 × 10^4 ^cells/cm^2 ^on cover slips or cover slips coated with either PLLA or PLLA/Col-I blend nanofibers in growth medium (DMEM), with low glucose and glutamine (PAA, Linz, Austria) supplemented with 10% FCS from selected lots (Stem Cell Technologies, Vancouver, Canada) and 1% penicillin/streptomycin. In order to facilitate the deposition of collagen, 0.05 mM ascorbic acid-2-phosphate was added to the medium. The medium was replaced every second day of culture during the experiments.

### Vitality staining

Vitality staining was performed using fluoreszein-diazetat (FDA). After 4 days of incubation, cover slips were removed from culture, rinsed with PBS and stained with FDA at a concentration of 5 μg/mL. Fluorescence microscopy was done using a Leica DM5000. Microphotographs of at least three different areas were made at a primary magnification of 20 fold high power field (HPF). Area of fluorescence was determined using Quips analysis software.

### Gene expression analysis

RNA was extracted from cell layers at days 4, 9 and 22 of culture using RNeasy Mini Kit (Qiagen GmbH, Hilden, Germany) according to the manufacturer and quantified spectrometrically. The cDNA was synthesized using Omniscript reverse transcriptase and oligo-dT primer in the presence of dNTP (Qiagen GmbH, Hilden, Germany). Quantitative RT-PCR was performed and monitored using a Mastercycler^® ^ep realplex Detection System (Eppendorf, Hamburg, Germany) and RealMaster Mix CyberGreen (Eppendorf, Hamburg, Germany). Genes of interest were analyzed in cDNA samples (5 μl for a total volume of 25 μl/reaction) using DeltaDeltaCt method and CyberGreen. Primers cycle temperatures and incubation times for collagens I, III and X as well as decorin and biglycan were published by Molloy et al. [26]. Focal adhesion kinase-1 (FAK) was analyzed using forward primer 5'ACC TCA GCT AGT GAC GTA TGG -3' and reverse primer 5'CGG AGT CCC AGG ACA CTG TG 3' (gen bank L0518666). For the protein-tyrosine kinases (PYK) analyzation, the following primers were used: forward 5'CAG CAG TAC GCC TCG CTC AG3' and reverse 5'TCA GCC TCT GCT AGG GAT GAG3' (gen bank U3328466). Phosphoinosytol-3-kinase (PI3K) was measured by using the forward primer 5'CCT GAT CTT CCT CGT GCTG CTC3' and reverse primer 3'ATG CCA ATG GAC AGT GTT CCT CTT5'. Cyclin D 1 (CCND) was analyzed using forward primers 5'ACG AAG GTC TGC GCG TGT T3' and reverse 5'CCG CTG GCC ATG AAC TAC CT3' (UniGene Hs.523852).

### Immunofluorescence microscopy of collagen I

Samples obtained at day 22 were fixed in aceton/methanol, washed with PBS (3×), and exposed to blocking buffer (1% donkey serum albumin PBS) for a further 30 min at room temperature in order to minimize non-specific absorption of the antibodies. After another wash in PBS (3×), the cells were incubated with primary antibodies against Col-I (Abcam, Ab6308, Cambridge, United Kingdom).

Visualization was done after washing in PBS (3×) using cy-3-conjugated secondary antibody (Dianova, Hamburg, Germany) at room temperature (1 hour). The slices were stained with DAPI (4.6-diamino-2-phenylindole) and embedded in mounting medium. Fluorescence microscopy was done using a Leica DM5000. Microphotographs of at least three different areas were made at a primary magnification of 20 HPF. Intensity of fluorescence was determined using Quips analysis software. Total cell count of DAPI stained nuclei, were obtained.

### Statistics

All values were expressed as mean ± standard error of three different patients analyzed at least in duplicate and compared using students' t-test or ANOVA with Bonferroni as a post hoc test. Values of p < 0.05 were considered to be significant.

## Results

### Characterisation of nanofiber scaffolds

**Figure 1:**
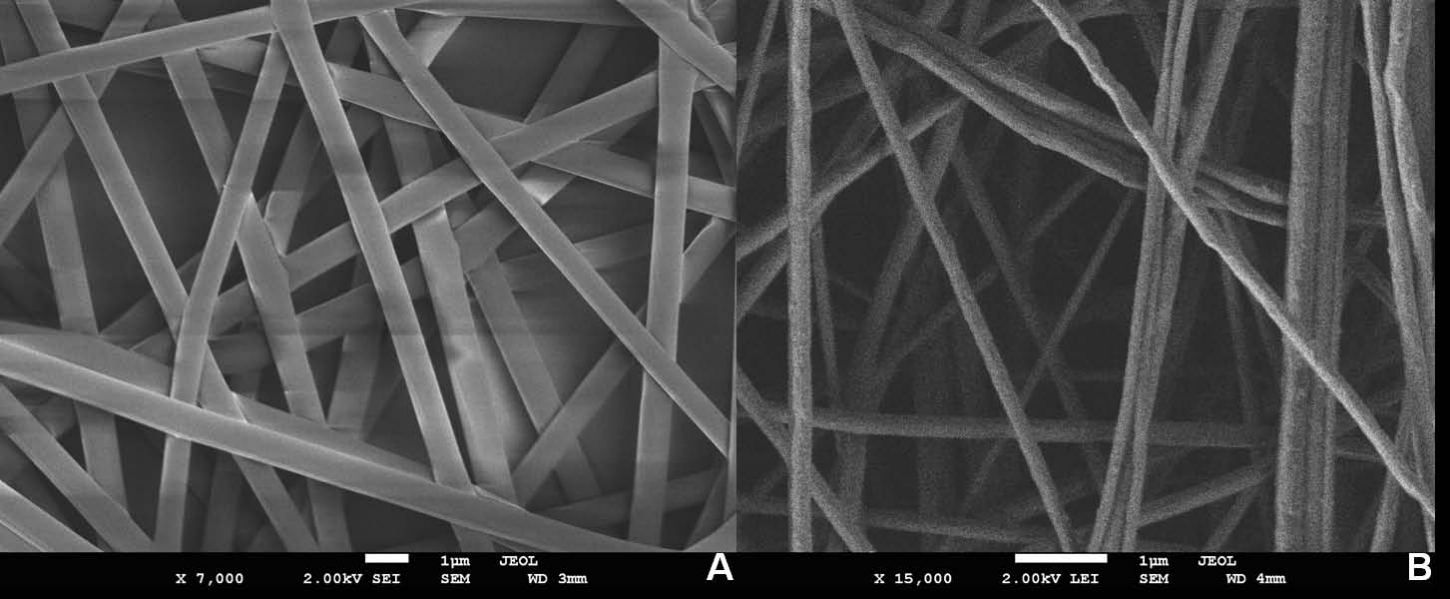
**Fiber characterization**. SEM analysis of PLLA (A) and PLLA/Col-I nanofibers (B).

Scanning electron microscopy PLLA nanofibers revealed a 3-D non-woven network with a mean diameter of 0.754 ± 295 μm. Fibers were smooth in structure (figure 1A), and had a contact angle of 118.4°. In contrast, blending with collagen I resulted in a decrease in fibre diameter (0.238 ± 93 μm) (figure 1B). Furthermore, the fibre scaffolds were more hydrophilic with a contact angle of less then 30°.

### Influence of PLLA and PLLA/Col-I nanofibers on cell vitality and proliferation

**Figure 2:**
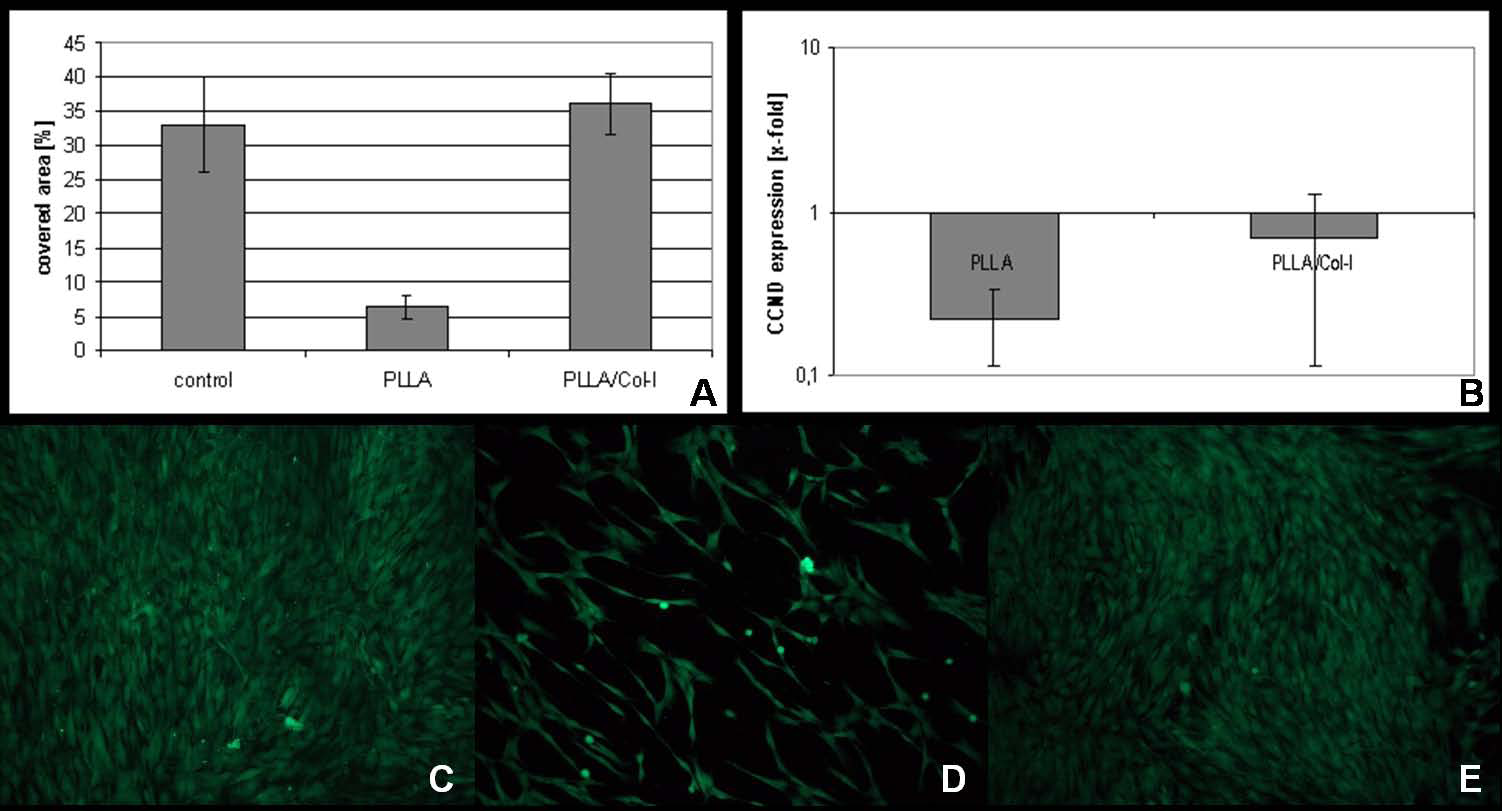
**Influence of PLLA and PLLA/Col-I nanofibers on growth of TDF's**. Area of FDA positive cells cultured over a period of 4 days (A) and relative CCND expression (B). Immunofluorescence microphotographs of FDA staining of TDF's cultured on glass (C), PLLA (D) and PLLA/Col-I (E) nanofiber scaffolds.

In order to describe the biological effects, we first analyzed the effect of PLLA and PLLA/Col-I blend nanofibers scaffold on the vitality of TDF's. As shown in figure 2, the presence of PLLA/Col-I nanofibers had no effect on cell densities after 4 days of cultivation (p = 1.000). On the other hand, significantly less living cells were detected on PLLA nanofibers at the same time (p = 0.001). This was accompanied by a down regulation of CCND (cyklin1D) gene expression (p = 0.001). CCND promotes progression through the G1-S phase of the cell cycle.

### Influence of PLLA and PLLA/Col-I nanofibers on matrix formation of hMSC

**Figure 3:**
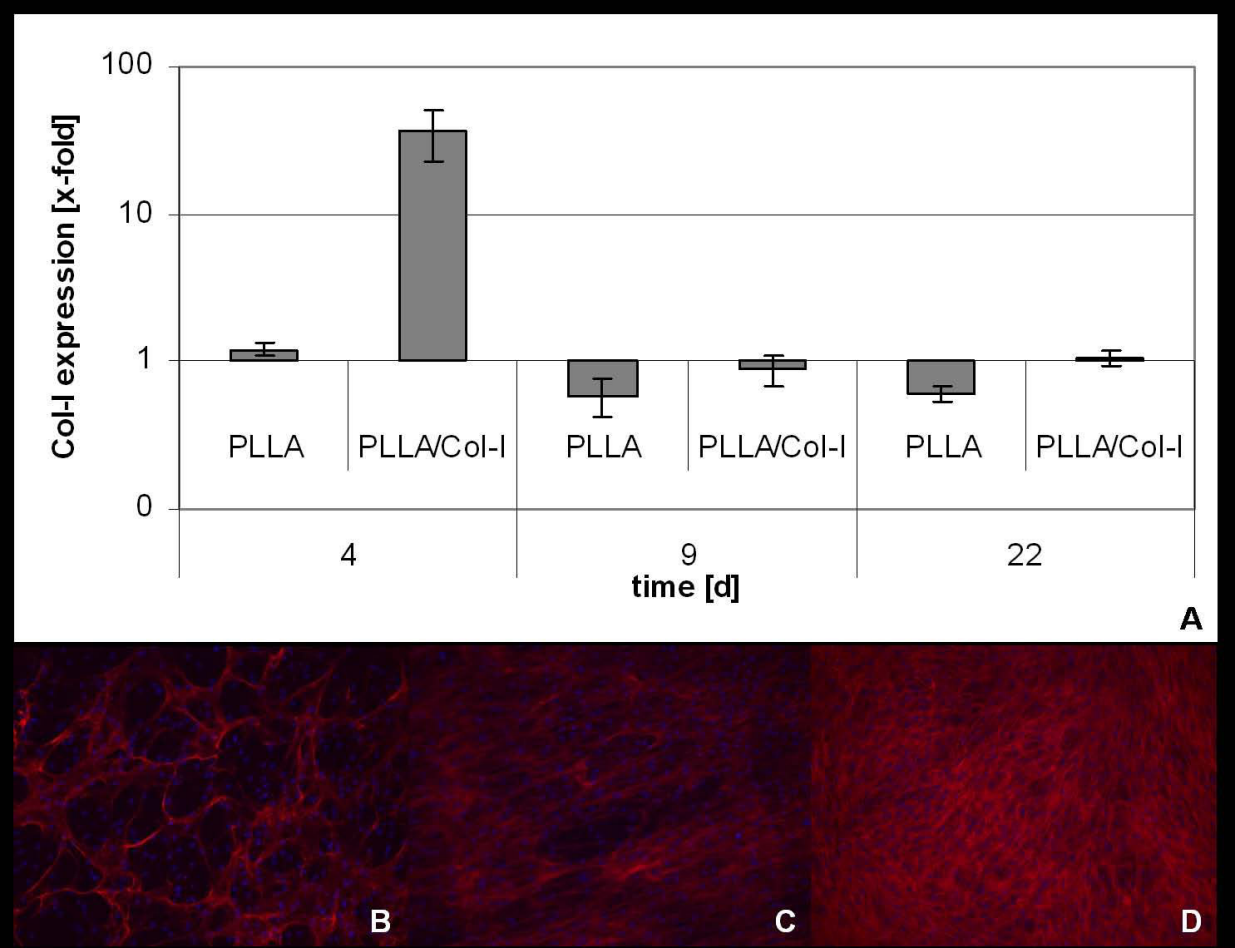
**Influence of PLLA and PLLA/Col-I nanofibers on gene expression Col-I deposition of TDF's**. Time course of collagen-I gene expression of TDF's on nanofibers compared to cover slips control (A). Immunofluorescence microphotographs of Col-I (red) deposition after 22 days of culture cover slip control (B), PLLA nanofibers (C) and PLLA/Col-I nanofibers (D).

Fibroblast production and deposition of type I collagen of TDF's cultured on PLLA and PLLA/Col-I blend nanofiber scaffolds was evaluated using time dependent gene expression analysis after 4, 9 and 22 days of cultivation as well as immunofluorescence analysis after 22 days of culture. As shown in figure 3, the presence of PLLA nanofibers had little effect on the expression of collagen I gene compared to cover slip control. Consequently, immunostaining of collagen I was similar to that obtained on cover slip control. Although there was a broad inter patient variability, the expression of collagen-I increased about 37 times compared to controls when TDF's were cultured in the presence of PLLA/Col-I blend nanofibers (p = 0.009). Although this increase was not stable over time, immunostaining of collagen I was more compact in structure and more intense in fluorescence compared to PLLA scaffolds or cover slip control when analyzed after 22 days.

Although the PLLA nanofibers did not influence the expression of collagen I gene, the presence of PLLA had a notable impact on the deposition of collagen. While TDF's on glass deposited the collagen in fiber bundles, on nanofibers the distribution was more equal (figure 3B-D) and similar to that obtained on PLLA/Col-I blend nanofibers.

**Figure 4:**
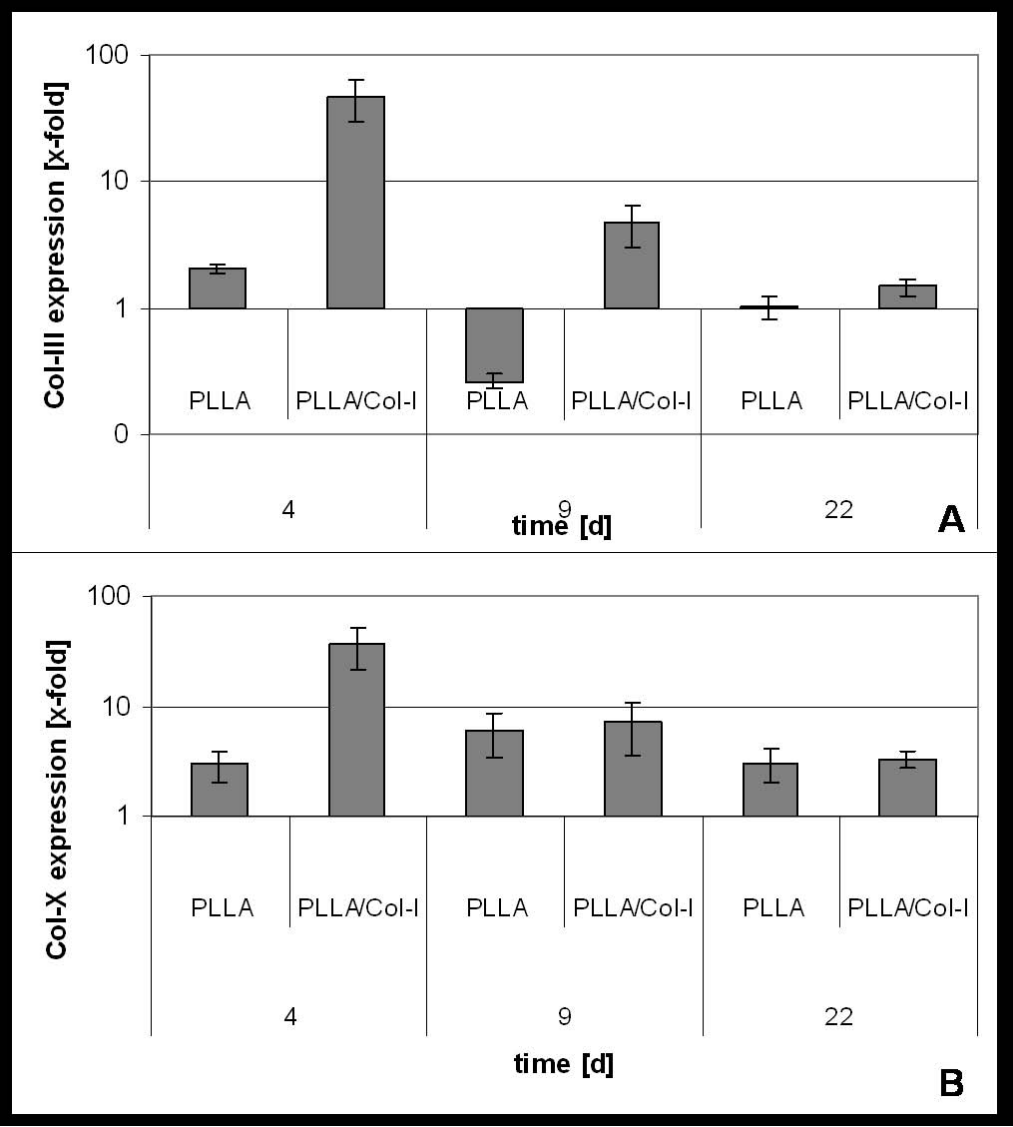
**Influence of PLLA and PLLA/Col-I nanofibers on gene expression Col-I deposition of TDF**. Time course of collagen-III (A) and collagen-X (B) gene expression of TDF's cultured on PLLA and PLLA/Col-I nanofibers as compared to cover slips control.

Due to the fact that during tendon repair, besides collagen I, the collagens III and X have been reported to play an important role and their gene expression was analyzed in a time dependent manner as well (figure 4). Similar to the gene expression of collagen I, the presence of PLLA nanofibers had only little effect on the collagen III gene expression, while the presence of PLLA/Col-I blend nanofibers resulted in a 5 to 100 fold increase depending on the cells (p = 0.009). Furthermore, this increase was prolonged and detectable over the whole period of culture (p = 0.038, day 9; p = 0.682, day 22).

Focusing on the gene expression of collagen X, both the presence of PLLA as well as PLLA/Col-I nanofibers resulted in a stable up regulation of the observed time. However statistical significance was reached only in case of PLLA/Col-I nanofibers at day 4 of cultivation (p = 0.021).

**Figure 5:**
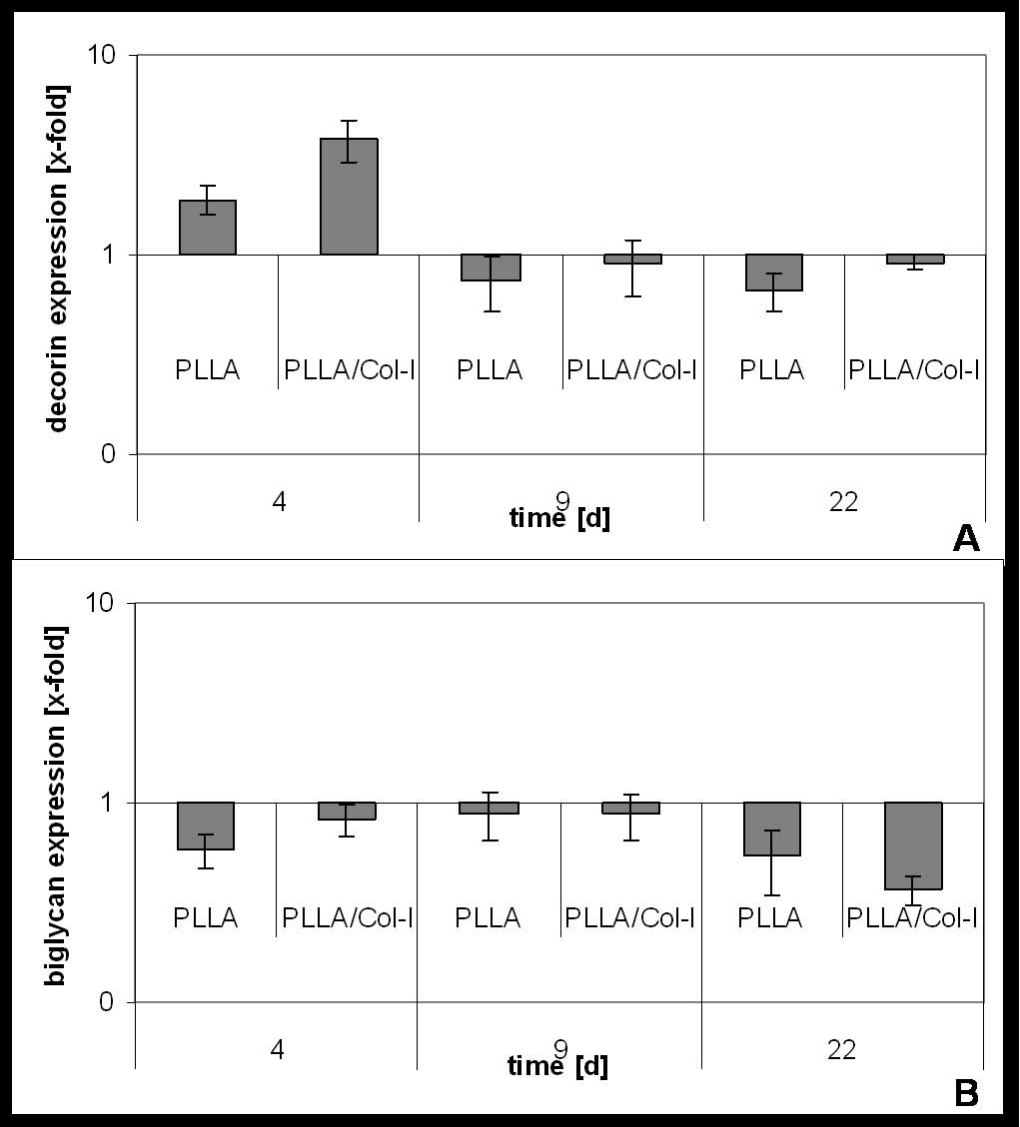
**Influence of PLLA and PLLA/Col-I nanofibers on gene expression proteoglycanes**. Time course of decorin (A) and biglycan (B) gene expression of TDF's cultured on PLLA and PLLA/Col-I nanofibers as compared to cover slips control.

Due to the fact that besides collagen, glucosaminoglycanes play an important role in tendon formation and function, we analyzed the gene expression of decorin and biglycan. As shown in figure 5, gene expression of decorin showed a comparable pattern to the expression of collagen I. Here, the initial increase on PLLA nanofibers was about 2 fold and on PLLA/Col-I nanofibers 4 fold, with statistical significance in case of PLLA/Col-I (p = 0.010). Focusing on the expression of biglycan, different results were obtained. The presence of nanofibers down regulated the gene expression irrespective of the nanofiber polymer composition. Significance was reached only during late time of cultivation (PLLA nanofibers: p = 0.031, day 9; p = 0.030, day 22; PLLA/Col-I nanofibers: p = 0.004, day 22).

### Influence of PLLA and PLLA/Col-I nanofibers on genes associated with the integrin signalling pathway

**Figure 6:**
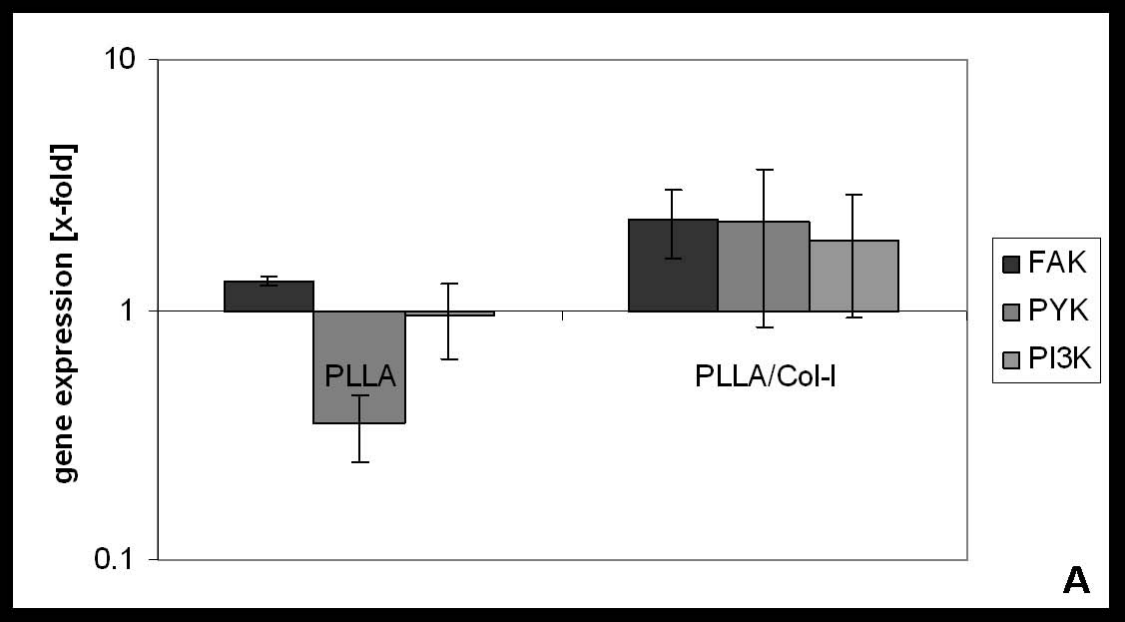
**Influence of PLLA and PLLA/Col-I nanofibers on gene expression genes involved in integrin signalling**. Gene expression of FAK, PYK and PI3K after 4 days of incubation PLLA and PLLA/Col-I as compared to cover slip control.

Because elevated expression of integrins has been associated with healing tendons [27] and ligaments [28] it is likely that this signalling pathway is involved in the effects elicited by nanofiber scaffolds - especially when blended with collagen. Indeed, when cultured on these nanofiber scaffolds we found an increased expression of FAK, PYK and phosphoinositide 3-kinase (PI3K) gene (figure 6) compared to PLLA scaffolds or cover slip control. However we found no significance due to a broad inter-patient variability.

## Discussion

PLLA is a biocompatible, biodegradable and by the Food and Drug Administration approved polymer [29,30]. In bone reconstructive surgery it is commonly used in pins, screws or membranes [31-34]. With respect to tendon repair, Koh et al. used PLLA patches to repair infraspinatus tears in a sheep model with good results [35]. When electrospun to nanofibers, these scaffolds were stable over a period of 30 days in aqueous solutions [25,36] or in the presence of cells - although there was some loss in maximum load and strain [36].

Furthermore, several cell types like neural stem cells (NSCs) [37,38], osteoblast like cell lines [39-41], endothelial cells [42] or mesenchymal stem cells [36] can be cultured on PLLA nanofibers.

In this study we first examined the ability of TDF's to grow on PLLA nanofiber scaffolds. Compared to glass surfaces, a significantly reduced number of cells were found. One reason might be that the high hydrophobic surfaces of PLLA nanofibers prevent the cell attachment. However, gene expression of CCND at this time point decreased indicating that PLLA nanofibers influence the proliferation of TDF's. This suggestion is supported by earlier studies using hMSC, where a decrease in monoclonal antibody Ki67 positive cells was detected compared to glass surfaces [43,44].

This inhibitory effect of PLLA polymer [45,46] and PLLA nanofibers [12] on cell densities was equalized when PLLA was blended with collagen or gelatine. As described for hMSC [22,47] we found no differences in cell densities of TDF's. This finding was accompanied by a normalisation of CCND expression. If so, this might indicate that the collagen component equalized the negative effects of PLLA on proliferation.

With respect to tendon replacement, besides growth, the formation of an extra cellular matrix plays an important role. Therefore, an ideal scaffold should support the formation of collagen I, the main component of the tendon, which is responsible for tensile strength [37,48,49]. When TDF's were cultured on PLLA/Col-I nanofibers, gene expression as well as the deposition of collagen I increased while the use of PLLA nanofibers alone had no or only minimal effect. These findings support earlier studies showing that PLLA/Col-I blend nanofibers increase the collagen expression in hMSC [22].

However, it is notable that the PLLA nanofibers influence the pattern of collagen deposition. The reason for this is unclear but it can be speculated that there is a link to the integrin pathway as seen in the expression of integrin in osteoblasts or stem cells on different nanofibers [50].

Within this context, the collagen integrin signalling may play an important role. Although not significant is that - due to broad inter-patient variability-TDF's expressed FAK, PYK and PI3K in higher levels when cultured on PLLA/Col-I blend nanofibers compared to PLLA nanofibers or glass surfaces. However, a part of this effect could be imputed to the nano-structured scaffold itself. In TDF's [51] as well as in other cell types, like osteoblasts [27], this effect was linked to increased *α*2 and *β*1 as well as *α*v and *β*3 integrins and an up regulation of phospho-paxillin and phospho-FAK in cell lysates compared to solid surfaces.

However, besides collagen I, other components of the extra cellular matrix are important for a proper tendon repair. With respect to scar formation, the collagens III and X have been reported to play an important role [52]. Especially collagen III is expressed in higher amounts during tendon healing. This has implications for the stability of repaired tendons [53,54]. Taking this for granted, the PLLA/Col-I blend nanofibers have to be seen critically due to their prolonged increase in collagen III expression. Here, further studies are needed in order to clarify whether this effect is compensated by the collagen I production.

As well as these aspects, tendon formation depends on the presence of proteoglycanes [55,56] which are involved in the formation of collagen fibrils, especially in the fibril diameter [49]. The different deposition pattern of collagen observed on nanofiber scaffolds might be associated with a down regulation of biglycan and may result in weaker tendons. However, the interaction between collagen I and biglycan is not yet completely understood [57]. Therefore further studies were needed in order to elucidate the interaction of nanofiber scaffolds and matrix formation.

## Conclusion

Taken together, this study indicates that the use of nanofibers influences the gene expression of genes associated with the extra cellular matrix formation. Here, the composition of the nanofiber plays a critical role. While PLLA/Col-I blend nanofibers enhance the collagen I and III formation, their expression on PLLA nanofibers was more comparable to controls. However, irrespective of the chemical composition of the fibres, the collagen deposition was altered, an effect which might be associated with a decreased expression of biglycanes.

## Authors' contributions

CT drafted the manuscript and was responsible for formulation of the manuscript as well as for data interpretation. CT, KK and JRJP were responsible for the study design and performing of the experiments. MDS was involved in drafting the manuscript. JRJP did the statistical analysis of the study and was responsible for the statistical methods used in the study. TE and JS took part in the formulation of the study and were also involved in drafting the manuscript. SFW participated in the description of background knowledge. All authors critically read, revised and finally approved the manuscript.

## Conflict of interests

The authors declare that they have no competing interests.
